# Misfolded Proteins and Cognitive Decline: Mechanistic Insights into Neurodegenerative Disorders

**DOI:** 10.3390/neurolint18030048

**Published:** 2026-03-02

**Authors:** Elisa Duranti, Chiara Villa

**Affiliations:** School of Medicine and Surgery, University of Milano-Bicocca, 20900 Monza, Italy; e.duranti@campus.unimib.it

**Keywords:** neurodegenerative diseases, cognitive disorders, protein aggregates

## Abstract

Cognitive decline represents one of the most common clinical manifestations of neurodegenerative diseases (NDs), substantially affecting the quality of life of both patients and their families. Alzheimer’s disease, Parkinson’s disease, and amyotrophic lateral sclerosis are major NDs characterized by a progressive degeneration of the central nervous system, with functional impairments extending beyond motor symptoms to multiple cognitive domains, including memory, attention, language, and executive functions. Increasing evidence highlights misfolded protein accumulation as a key driver of neuronal dysfunction and cognitive deterioration. This narrative review examines the major cognitive deficits associated with these disorders, focusing on the underlying molecular mechanisms, particularly protein aggregation, as well as clinical manifestations and their effects on daily life. Furthermore, current diagnostic tools and emerging therapeutic options for mitigating cognitive decline will be further discussed.

## 1. Introduction

Cognitive impairment is a central and progressively disabling feature of many neurodegenerative disorders (NDs), including Alzheimer’s disease (AD), Parkinson’s disease (PD), and amyotrophic lateral sclerosis (ALS), profoundly affecting patients’ quality of life. These deficits not only burden patients but also place a substantial emotional and economic burden on caregivers and healthcare systems [1]. These NDs share common pathogenic mechanisms, including progressive neuronal loss, synaptic dysfunction, and widespread brain atrophy, which collectively contribute to cognitive deterioration [2,3]. While each of these NDs has a distinct clinical presentation and etiology, cognitive impairment emerges as a common and often devastating hallmark, particularly in advanced stages when patients lose autonomy in daily activities [4,5]. The course of cognitive deterioration differs markedly across NDs. For example, episodic memory loss is a defining early hallmark in AD, followed by difficulties with language, spatial orientation, and executive functions as the disease advances [6]. In contrast, PD is initially characterized by motor dysfunction, but cognitive impairments, particularly in executive functions, working memory, and visuospatial abilities, become more apparent as the disease progresses [7]. ALS, which was historically believed to be a motor neuron disease, is also associated with cognitive dysfunction, notably in the domain of executive control, with up to 50% of patients developing some degree of cognitive impairment [8]. A subgroup of ALS patients develops a full-blown dementia syndrome overlapping with frontotemporal dementia (FTD-ALS), which complicates both prognosis and management [9].

At the molecular level, a unifying pathological hallmark is the abnormal accumulation of misfolded proteins, which plays a pivotal role in driving neuronal dysfunction and disease progression [3]. Disease-specific protein aggregates characterize each ND: amyloid-β (Aβ) plaques and tau neurofibrillary tangles (NFTs) in AD [10,11], α-synuclein (α-syn) inclusions forming Lewy bodies in PD [12,13], and pathogenic proteins such as superoxide dismutase 1 (SOD1), TAR DNA-binding protein 43 (TDP-43), fused in sarcoma/translocated in liposarcoma (FUS/TLS), and chromosome 9 open reading frame 72 (C9ORF72) in ALS [2,14]. Despite the fact that these proteins differ in structure and physiological function, they converge on a common pathogenic cascade driven by protein misfolding, impaired proteostasis, and defective clearance mechanisms [15]. The progressive accumulation of toxic protein species leads to synaptic dysfunction, mitochondrial impairment, oxidative stress, and chronic neuroinflammation [16]. These processes ultimately result in the collapse of the structure and function of neuronal circuits, leading to deficits in memory, behavior, executive functions, language, and visuospatial processing [17]. Importantly, growing evidence indicates that the pattern and regional distribution of misfolded protein aggregates determines the clinical expression of cognitive symptoms [18]. For instance, the early hippocampal deposition of Aβ and tau underlies memory impairment in AD [18,19,20] whereas α-syn aggregation is more abundant in the anterior cingulate of PD patients with cognitive impairment [21]. In ALS, the pathological accumulation of TDP-43 in cortical brain regions is strongly associated with motor and cognitive function [22].

Collectively, these findings support the hypothesis that misfolded proteins play a crucial role in cognitive decline across NDs. Early diagnosis of cognitive impairment is essential for optimizing personalized treatment strategies and improving patient outcomes. Therefore, the purpose of this review is to explore the major cognitive deficits associated with NDs, focusing on the underlying molecular mechanisms, particularly protein aggregation. Moreover, available diagnostic tools and novel therapeutic options targeting cognitive decline will be also discussed.

## 2. Cognitive Impairments in Neurodegenerative Diseases: A Mechanistic Perspective

Cognitive impairments represent a common feature of NDs, resulting from multiple interconnected molecular and cellular mechanisms. Rather than being attributable to a single pathological event, cognitive decline reflects the combined effect of protein misfolding and aggregation, impaired proteostasis, chronic neuroinflammatory responses, synaptic dysfunction, neuronal loss, and metabolic alterations. This section offers a mechanistic approach to provide a comprehensive framework for understanding how these processes contribute to neuronal dysfunction and cognitive deficits.

### 2.1. Comprehensive and General Insight into Neurological Protein Aggregates

NDs are increasingly recognized to have overlapping cellular and molecular processes, including protein aggregation and the development of inclusion deposits [23]. Under normal conditions, newly synthetized proteins achieve and maintain their correct conformation through cellular protein quality control (PQC) mechanisms that ensure proteostasis [24]. When these processes are impaired, proteins can become unstable and prone to aggregate. Protein misfolding and aggregation can be triggered by genetic mutations, aging, environmental damage, alterations in the cellular mechanisms that regulate PQC, changes in the intracellular environment such as temperature shifts, pH changes, oxidative stress, and the presence of metal ions [25,26]. These protein aggregates can interfere with essential cellular functions by altering intracellular signaling, inducing oxidative stress, and promoting neuronal death [27]. In response, neurons activate stress-response mechanisms such as molecular chaperones and degradation machinery through the ubiquitin–proteasome system (UPS) and autophagy–lysosomal pathway (ALP) [28]. However, in NDs, these protective mechanisms are not sufficient to manage the accumulation of protein aggregates, establishing a vicious cycle of neuronal damage and cell death. Molecular chaperones are also vital players in the cellular PQC network, crucial for checking the proteins machinery, especially in long-lived cells like neurons that do not proliferate [23]. The CNS is especially vulnerable to proteotoxic stress because it requires high energy consumption and a restricted ability to regenerate and is constantly faced with oxidative and inflammatory stressors. In this scenario, heat shock proteins (Hsps) such as Hsp40 (from the DNAJ family), Hsp60, Hsp70, Hsp90, and small heat shock proteins (sHsps) act as the first line of defense against proteins that misfold or aggregate together [29,30]. Chaperones contribute in the proper folding of new polypeptides, the refolding of stress-damaged proteins, the prevention of undesired protein interactions, and the choice of whether a protein should be refolded or degraded [31]. This selection process is carefully controlled and involves constant communication with the UPS and the ALP [23,32]. When the chaperones become overloaded or do not work properly, misfolded proteins can accumulate, leading to aggregation, synapse failure, and eventually, neuronal death [23]. Aging represents the biggest risk factor for many NDs and is associated with a gradual decline in chaperone levels and their activation and function [33]. According to the research, it has been shown that the heat shock response decreases with age, resulting in a reduced activation of protective chaperones when cells are under stress. This decline significantly reduces the susceptibility of neurons to proteotoxic damage, allowing disease-related protein aggregates to accumulate [34,35].

Under normal physiological settings, enzymatic post-translational modifications (PTMs) also represent tightly regulated mechanisms aimed to enhance the localization, function, and interactions of proteins [36,37]. However, this control becomes lost in disease states or under cellular stress, frequently due to the dysfunction of the upstream signaling pathways that regulate these modifying enzymes. Loss of balance can lead to aberrant or excessive PTMs, which can change the protein structure and increase its tendency to misfold and aggregate [37].

### 2.2. Disease-Specific Pathological Proteins

#### 2.2.1. Aβ in Alzheimer’s Disease

The Aβ protein is related with the formation of senile plaques in the brain, a key hallmark of AD pathology [38]. Aβ is derived from the sequential cleavage of the amyloid precursor protein (APP) by β-secretase and γ-secretase enzymes, resulting in Aβ peptides of varying sizes, namely Aβ38, Aβ40, and Aβ42 [39]. Among these, the Aβ42 isoform is more likely to misfold and aggregate, producing cytotoxic prefibrillar oligomers and fibrils that accumulate in the brain [40]. Aβ can undergo various PTMs, including phosphorylation, oxidation, glycosylation, nitration, isomerization, and pyroglutamylation, leading to peptides with different physiological or pathological properties [41].

#### 2.2.2. Tau in Alzheimer’s Disease

Tau is a microtubule-associated protein required for cytoskeletal structural stability and involved in key signaling pathways [42,43]. In the human brain, six isoforms result from alternative splicing of the exons 2, 3, and 10 of the microtubule associated protein tau (*MAPT*) gene [44]. The alternative splicing of exon 10 generates tau isoforms with three (3R) or four (4R) microtubule-binding repeats found in equal amounts in the adult human brain and required for normal function [45]. An imbalance in the 3R:4R tau ratio leads to neuronal dysfunction and is linked to NDs [46]. Tau also undergoes PTMs, with phosphorylation being the most significant. Hyperphosphorylated tau loses microtubule affinity, leading to cytoskeletal destabilization and NFT formation [47]. Tau can evade PQC mechanisms through multiple strategies: hyperphosphorylation impairs recognition by E3 ubiquitin ligases, preventing degradation; tau aggregates disrupt autophagosome formation, limiting their clearance; misfolded tau forms β-sheet–rich structures that are resistant to proteolysis; aberrant interactions with stress granules promote tau stabilization and aggregation via phase separation [48].

#### 2.2.3. Alpha-Syn in Parkinson’s Disease

Alpha-syn plays a key role in PD and is highly expressed in presynaptic neurons [49]. While its exact function remains unclear, it is involved in synaptic vesicle regulation. A major pathological feature of PD is the accumulation of insoluble α-syn aggregates, known as Lewy bodies, which impair neuronal function and drive neurodegeneration [50,51]. Its aggregation and stability are influenced by various PTMs, including phosphorylation, ubiquitination, truncation, and acetylation [52].

#### 2.2.4. SOD1 in Amyotrophic Lateral Sclerosis

Mutations in the *SOD1* gene on chromosome 21 were linked to ALS. This gene encodes the Cu/Zn SOD1 enzyme, which plays a crucial role in the cytoplasm by converting superoxide radicals into hydrogen peroxide and molecular oxygen [53]. SOD1 is widely expressed, is highly conserved, and makes up about 1% of the total protein content in the cytoplasm of cells [54,55]. Its function and stability are regulated through various PTMs on key amino acid residues, such as phosphorylation, lysine modifications, redox changes, and nitration [56].

#### 2.2.5. TDP-43 and FUS in Amyotrophic Lateral Sclerosis

TDP-43 is a protein of considerable interest due to its high degree of conservation across different species and its widespread presence in both human and rodent cells, where it is primarily located in the nucleus. This protein, made up of 414 amino acids and weighing 43 kDa, is encoded by the *TARDBP* gene on chromosome 1. It is a member of the heterogeneous ribonucleoprotein family, a diverse group of RNA-binding proteins [57,58]. The abnormal aggregation and mislocalization of TDP-43 are linked to a range of PTMs that can alter its activity and cellular behavior. These include phosphorylation, C-terminal fragment formation, disulfide bond creation, acetylation, ubiquitination, and SUMOylation, which have been the focus of extensive study [59]. Under normal conditions, TDP-43 plays roles in RNA regulation, including transcriptional regulation, alternative splicing, and mRNA stabilization [2,60]. However, in ALS, TDP-43 undergoes abnormal phosphorylation, mislocalization, and aggregation, leading to the formation of cytoplasmic inclusions in affected neurons [61,62,63]. These pathological changes interfere with normal cellular functions, contributing to the degeneration of motor neurons. A major role of TDP-43 has recently emerged as a repressor of cryptic exons during splicing [64]. Unlike normal conserved exons, cryptic exons reside in introns of genes and are normally excluded from mature messenger mRNAs (mRNAs) [64,65]. When TDP-43 is depleted from the nucleus in ALS, these cryptic exons are spliced into mRNAs, often introducing frame shifts and premature termination, preventing the expression of crucial proteins including STMN2 and UNC13A [2,66,67,68].

The identification of TDP-43 mutations in ALS paved the way to the discovery of mutations in a second RNA/DNA-binding protein, FUS/TLS, which accounts for 4–5% of familial ALS (fALS) and 1% of sporadic ALS (sALS), particularly with early onset and short survival [69]. FUS is widely expressed in human tissues, primarily in the nucleus, but it also shuttles to the cytoplasm, where it participates in processes like DNA repair, transcription regulation, splicing, and mRNA transport and maturation [70]. In the central nervous system (CNS), FUS promotes mRNA transport to dendrites and synaptic plasticity following glutamate receptor activation [71]. Most pathogenic FUS mutations affect the C-terminal region, specifically the nuclear localization signal (NLS), disrupting its interaction with transportin-1 and the nuclear-cytoplasmic balance [71].

#### 2.2.6. C9ORF72 in Amyotrophic Lateral Sclerosis

The expansion of the intronic hexanucleotide repeat (GGGGCC) in *C9ORF72* gene represents the most commone genetic cause of ALS [72,73], leading to the formation of aggregates through different mechanisms [74]. The expanded repeat forms RNA foci and undergoes repeat-associated non-AUG translation, producing toxic dipeptide repeat proteins. These ones accumulate in the nucleus and cytoplasm, where they impair PQC, nucleocytoplasmic transport, and stress granule dynamics, all of which contribute to neurodegeneration [74].

### 2.3. Pathophysiological Convergence: From Protein Misfolding to Cognitive Decline

**Figure 1:**
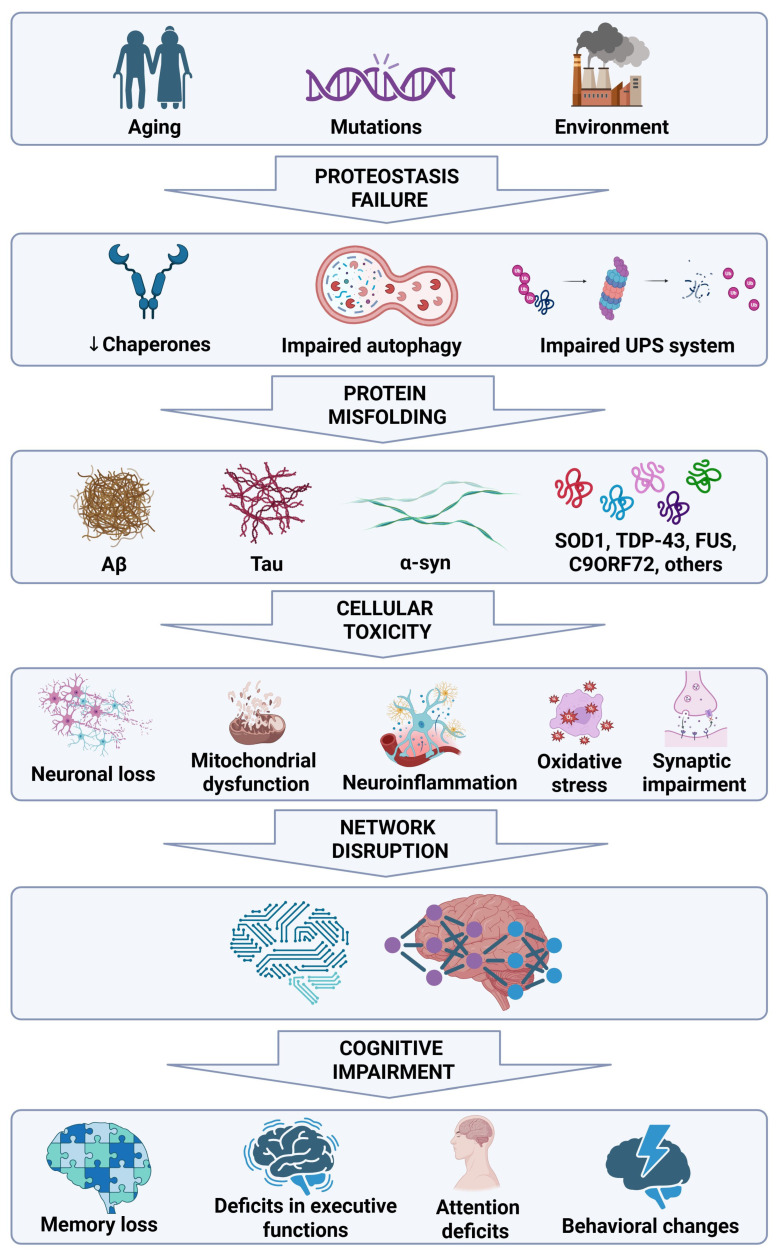
**Pathogenic cascade linking protein misfolding to neurodegeneration.** Protein misfolding triggers a multistep pathogenic process that leads neuronal loss, mitochondrial dysfunction, neuroinflammation, oxidative stress, and synaptic impairment. These interconnected mechanisms progressively disrupt neuronal function and network integrity, ultimately resulting in neurodegeneration and cognitive impairment. Created with BioRender.com. Villa, C. (2026) https://BioRender.com/we2emft (accessed on 24 February 2026).

Despite the molecular heterogeneity of these different NDs, emerging evidence indicates that protein misfolding represents an initiating event rather than a disease-specific hallmark, triggering a cascade of downstream interconnected biological processes that eventually impair cognitive function [15] (Figure 1).

**Figure 2:**
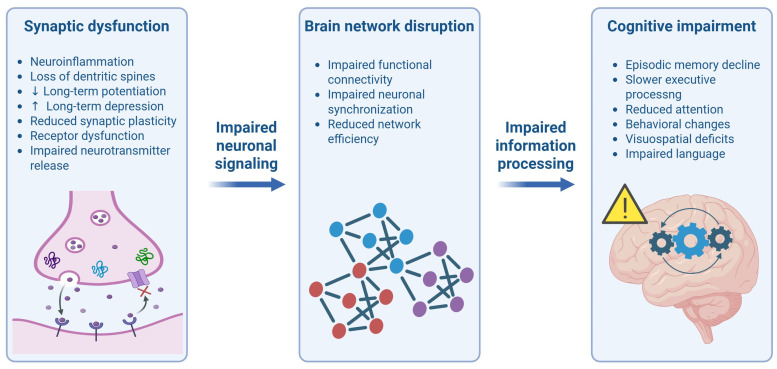
**Multiscale framework linking early synaptic dysfunction to large-scale network alterations and subsequent cognitive decline.** At the synaptic level, neuroinflammation, loss of dendritic spines, decreased LTP, increased LTD, reduced synaptic plasticity, receptor dysfunction and impaired neurotransmitter release disrupt neuronal signaling. These local changes propagate to the network level, leading to altered functional connectivity, impaired synchronization, and reduced efficiency. Network reorganization ultimately compromises information processing, contributing to cognitive impairment, including episodic memory decline, reduced attention, behavioral changes, visuospatial deficits and impaired language. Created with BioRender.com. Villa, C. (2026) https://BioRender.com/y79usib (accessed on 24 February 2026).

In NDs, protein misfolding arises from a progressive failure of cellular proteostasis mechanisms, driven by aging, genetic susceptibility and impaired PQC systems, resulting in the deposition of aggregation-prone proteins in different brain regions where they exert toxic effects on neuronal structure and function [15]. These species appear to be particularly increased during the early pathological stages and preferentially accumulate at synapses in vulnerable brain regions, where they disrupt synaptic structure and function, leading to synaptic loss, reduced synaptic density, and impairments in neurotransmitter release, synaptic plasticity, and receptor trafficking [75,76]. As a result, synaptic dysfunction occurs before overt neuronal death, indicating a higher susceptibility of synaptic compartments to proteotoxic and metabolic stress. This synaptic failure ultimately manifests as altered neuronal connectivity within large-scale cognitive networks, including hippocampal, fronto-parietal, and cortico-striatal circuits [77,78,79]. Functional disconnection within these networks offers a mechanistic explanation for why different NDs converge on similar cognitive phenotypes, such as attention, memory, and executive function deficiencies, since cognition arises from distributed network activity [75] (Figure 2).

In addition to protein misfolding-induced synaptic dysfunction, multiple biological processes concomitantly contribute to cognitive decline in NDs. Among these, neuroinflammation plays a pivotal role in disease progression. Notably, misfolded protein aggregates can act as damage-associated molecular patterns (DAMPs) and activating inflammasomes, hence stimulating innate immune signaling and neuroinflammatory responses [80,81]. Microglia, the resident immune cells of the CNS, normally maintain brain homeostasis by clearing debris and responding to damage [82]; however, under pathological conditions, chronic microglia activation promotes the release of pro-inflammatory phenotype, releasing reactive oxygen species (ROS) and a cascade of cytokines such as IL-1β, TNF-α, and IL-6 [83]. Similarly, astrocytes represent another cell population involved in the inflammatory response through the release of different pro-inflammatory mediators. A change in their shape, abundance, and activity is frequently observed in NDs [84]. Astrocytes may contribute to the progression of NDs by losing homeostatic function at the synapse and gaining a reactive phenotype, known as astrogliosis in response to protein aggregate accumulation, which often occurs in parallel to loss of synapses [85]. This persistent inflammatory environment exacerbates neuronal damage and synaptic dysfunction by altering neurotransmitter balance, disrupting synaptic pruning, and promoting neuronal stress, thereby contributing to cognitive impairment [86].

Neuronal metabolism is crucial for maintaining synaptic transmission, plasticity, and overall cognitive function. Because neurons generate adenosine triphosphate (ATP) nearly exclusively by oxidative phosphorylation, even small metabolic alterations can have a significant impact on synaptic transmission and plasticity [87]. Mitochondrial dysfunction affects ATP synthesis, calcium buffering, and mitochondrial dynamics, reducing the metabolic reserve required to maintain synaptic activity and function. These changes are strongly associated with the increased production of ROS, which promotes oxidative stress and further damages synaptic proteins, membranes, and signaling pathways [88,89,90]. Notably, defective mitochondrial biogenesis and mitophagy, as well as abnormal dynamics, are frequently observed in different models of NDs, even before the manifestation of pathological hallmarks, suggesting that mitochondrial dysfunction contributes not only to disease progression but also to its onset [85]. Mitochondrial DNA and other mitochondrial damage-associated molecular patterns (mtDAMPs) are released when cells are stressed or damaged and activate the innate immune responses [80]. Collectively, these observations strongly underline the intricate and self-perpetuating relationship between mitochondrial damage, neuroinflammation, and oxidative stress, which form a vicious cycle rather than acting as distinct processes. Mitochondrial malfunction triggers inflammatory responses that sustain the production of toxic mediators, such as pro-inflammatory cytokines and ROS. In turn, inflammatory signaling increases metabolic demand and mitochondrial stress, further impairing mitochondrial function and increasing ROS production. The resulting oxidative damage exacerbates glial inflammatory activation, ultimately enhancing neuroinflammation and promoting progressive neuronal death [91].

Together, all these processes disrupt calcium homeostasis, impair proteostasis, and compromise the structural and functional integrity of synapses. By progressively weakening synaptic transmission and large-scale network connections, they amplify the effects of protein misfolding and promote cognitive decline.

## 3. Cognitive Impairment in Major Neurodegenerative Diseases

This section provides an in-depth examination of the cognitive impairments associated with AD, PD, and ALS. For each condition, the relationship between cognitive characteristics and molecular mechanisms that underlie the development of disease will be investigated, offering a broad framework for understanding cognitive dysfunction in NDs.

### 3.1. Alzheimer’s Disease

#### 3.1.1. Cognitive Characteristics

AD is the leading cause of dementia worldwide, characterized by progressive cognitive decline, including impairments in memory, orientation, language, and abstract thinking. One of the earliest and most prominent clinical signs of AD is short-term memory loss, making it increasing difficult to store and retrieve new information [92,93]. Studies have shown that brain regions involved in episodic memory, such as the hippocampus and entorhinal cortex, are among the first affected [94]. As AD progresses, cognitive decline extends to semantic and autobiographical memory, significantly impacting functional abilities [95].

Other cognitive deficits include spatial and temporal disorientation, in which patients experience difficulty recognizing familiar places and managing time [96]. Language impairments, including word-finding difficulty and reduced sentence complexity, are also common and become progressively more evident as the disease advances [97,98]. Alterations in abstract thinking compromise the ability to plan, solve problems, and make decisions, further limiting their independence [99].

#### 3.1.2. Molecular Mechanisms Underlying Cognitive Impairments

Although Aβ and tau pathology define AD, cognitive impairment is increasingly recognized to arise from synaptic and network dysfunction rather than neuronal loss alone. The aberrant cleavage of APP generates toxic Aβ peptides that aggregate into plaques, mainly in the cerebral cortex and hippocampus, the brain areas associated with cognitive function decline in AD [43,100]. Aβ deposits disrupt synaptic communication and trigger neurotoxic events [101]. In parallel, tau protein undergoes abnormal hyperphosphorylation, leading to intracellular aggregation, impaired axonal transport [102,103], and progressive spreading from the hippocampus and entorhinal cortex to other cortical regions [104]. The combined effects of Aβ deposition and tau pathology exacerbate neurodegeneration and directly contribute to the cognitive decline observed in AD patients [20]. Notably, the accumulation of misfolded proteins can be also promoted by a dysfunction of cellular PQC systems [105]. In this regard, reduced levels and altered activity of molecular chaperones such as Hsp70 and Hsp90 have been observed in affected brain regions and correlate with increased Aβ and tau pathology [106]. Hsp70 is essential for maintaining tau solubility and preventing its harmful aggregation while Hsp90 aids in the stabilization of incorrectly folded tau proteins [107,108]. It has been reported that phosphorylation at specific tau residues (Ser202/Thr205) prevents its interaction with Hsp90, thereby stabilizing its toxic conformation [109].

Synaptic loss in the neocortex and hippocampus is consistently observed in AD and precedes neuronal death in several studies [77,110,111,112], representing an early driver of AD-related cognitive decline [110,113]. Consistently, changes in synaptic transmission frequently occur prior to the onset of tau disease, further supporting synaptic dysfunction as an initial pathogenic event [113]. Soluble forms of Aβ and tau are more harmful to synapses than their aggregated counterparts, mainly in the asymptomatic stages of the disease [114]. Soluble oligomeric Aβ disrupt synaptic transmission by impairing long-term potentiation (LTP) and promoting long-term depression (LTD), two critical mechanisms for learning and memory [115]. In parallel, tau hyperphosphorylation destabilizes microtubules, leading to synaptic collapse and neuronal death, particularly in the hippocampus, a region crucial for memory consolidation [116]. While Aβ plaque may damage neurons by interfering with neuron–neuron transmission at synapses, NFTs disrupt the intracellular transfer of nutrients within neurons, compromising synaptic integrity [117]. In addition to functional damage, the accumulation toxic Aβ also affects the shape and composition of synapses. Low levels of key pre- and postsynaptic proteins, including synaptosomal-associated protein 25 (SNAP-25), synaptophysin, synaptotagmin, and the dendritic spine structural protein drebrin, are found in AD patients [118,119]. Importantly, the idea that synaptic dysfunction is an early and crucial step in the disease pathogenesis is also supported by the fact that alterations in synaptic protein composition can occur before the deposition of Aβ plaque [110]. Neuroinflammation further amplifies these pathogenic processes: Aβ plaques in AD trigger microglial activation, which accelerates the deposition of both Aβ and tau. Activated microglia release ROS and pro-inflammatory cytokines, sustaining neuroinflammatory responses that promote tau hyperphosphorylation, oxidative stress, synaptic dysfunction, and mitochondrial impairment. Together, these processes exacerbate neuronal stress, accelerate neuronal death, and worsen cognitive decline [86]. In parallel, the activation of astrocytes further amplifies neuroinflammation, disrupting motor circuits in the motor cortex, basal ganglia, and cerebellum. This widespread neural damage contributes to motor symptoms such as gait disturbances and balance problems, highlighting the strong interconnection between cognitive and motor dysfunction in AD [120].

Mitochondrial abnormalities represent another major contributor to synaptic failure in AD [121]. Impaired mitochondrial dynamics and function, including imbalanced fission/fusion, reduced mitophagy, and abnormal mitochondrial trafficking, result in neuronal malfunction and degeneration associated with increased ROS production, decreased ATP generation, and disrupted intracellular calcium buffering [121,122]. These mitochondrial alterations have been frequently associated with the early stage of the disease [123,124] and ultimately lead to the typical loss of synapses in the hippocampus and neocortex of AD patients, which is linked to cognitive deficits such as in memory [121,125]. Moreover, reduced glucose metabolism in the brain is strongly correlated with cognitive decline in AD, further highlighting the role of bioenergetic failure in disease progression [126]. Importantly, mitochondrial impairment not only disrupts cellular energy metabolism but also contributes directly to the molecular pathology of AD. Some studies have proposed that mitochondrial dysfunction may lead to amyloid and tau deposition while Aβ itself interferes with mitochondrial transport and fission/fusion dynamics. In this respect, Aβ has the ability to enter mitochondria where it is processed and degraded by specific proteases, lowering its toxicity. This proteolytic activity is reduced in AD brains [127], likely due to increased ROS, reinforcing the connection between oxidative stress and Aβ processing [128].

### 3.2. Parkinson’s Disease

#### 3.2.1. Cognitive Characteristics

PD is primarily known as a movement disorder characterized by motor symptoms such as bradykinesia, rigidity, and tremors [129]. However, cognitive impairments are increasingly recognized as significant components of the disease, affecting the quality of life and functional independence [130]. Cognitive impairments in PD encompass a range of deficits that evolve throughout the disease course. Individuals with PD frequently experience various cognitive deficits that significantly impact their daily lives. Among these, attention deficits are particularly common, with patients frequently reporting difficulties in maintaining attention, which can hinder their ability to execute daily tasks and engage in complex activities. Such deficits are especially pronounced during multitasking scenarios, where the need to divide attention among multiple activities further exacerbates the issue [131,132,133]. Another cognitive feature of PD is slowness of thought. Patients may face difficulties in rapidly switching between tasks or retrieving information from memory, which can impact their overall cognitive performance, particularly in situations that require rapid decision-making or mental agility [134,135]. In addition to these cognitive deficits, many individuals with PD experience executive dysfunction, which refers to difficulties with higher-order cognitive processes like planning, problem-solving, and cognitive flexibility. These impairments can manifest as challenges in organizing thoughts, managing time effectively, and adapting to new situations [136,137]. Finally, visuospatial memory deficiencies are another common cognitive impairment in PD which affect the ability to process and interpret spatial and visual information, making it difficult to navigate situations or understand visual cues. Recognizing objects, evaluating distances, or orienting oneself in a familiar setting are all examples of tasks that require spatial awareness [138,139].

#### 3.2.2. Molecular Mechanisms Underlying Cognitive Impairments

The cognitive deficits observed in PD are intricately linked to a complex interplay of neurodegenerative and molecular mechanisms, as well as to disruptions in multiple neurotransmitter systems. The aggregation of misfolded α-syn into Lewy bodies causes neuronal toxicity and cell death, particularly affecting dopaminergic neurons of the substantia nigra [140,141]. This degeneration disrupts the dopaminergic pathways essential for both motor and cognitive functions. Accordingly, some studies have revealed that the degree and spread of α-syn pathology correlate with the severity of cognitive impairment, highlighting its role in the progression of non-motor symptoms in PD [142]. At the molecular level, cellular PQC systems attempt to counteract α-syn toxicity through molecular chaperones, such as Hsp70 and sHsps, which promote proper protein folding and facilitate the degradation of misfolded α-syn [143,144,145]. However, the failure of these protective mechanisms leads to α-syn accumulation, which in turn promotes microglial activation and neuroinflammation. These processes further exacerbates dopaminergic neuron damage in cognitive-related regions such as the prefrontal cortex [146,147].

Another significant contributor to cognitive deficits in PD is the dopaminergic depletion resulting from the degeneration of neurons in the substantia nigra. Beyond its role in motor control, dopamine plays a pivotal role in regulating executive functions and attention. Dopaminergic loss the prefrontal cortex and striatum can disrupt cortico-striatal synaptic circuits, leading to deficits in executive function, attention, and working memory [148,149]. Intriguingly, research indicates that cognitive deficits in PD may manifest even before the onset of pronounced motor symptoms, underscoring the importance of understanding the neuroanatomical changes associated with dopaminergic reduction [149]. In addition to dopaminergic alterations, the dysfunction of other neurotransmitter systems further contributes to cognitive decline in PD. In particular, the loss of cholinergic neurons in the basal forebrain, combined with low levels of acetylcholine, exacerbates cognitive impairments. This dysfunction particularly affects memory, attention, and learning processes, contributing to the multidimensional cognitive challenges faced by patients with PD [150].

Mitochondrial dysfunction acts as an amplifying mechanism in PD-related cognitive impairment. Similarly to AD, defects in the complex I of the electron transport chain lead to a reduced ATP synthesis and increased ROS generation, resulting in dopaminergic neuron loss and cognitive impairment [151,152,153]. Interestingly, distinct patterns of cognitive impairment are linked to the wide range of mechanisms that cause mitochondrial dysfunction, depending on the affected brain region [153]. Reduced ATP production in the hippocampus and prefrontal cortex is associated with memory deficits [154] while alterations in mitochondrial dynamics and morphology in the parietal and occipital cortex as well as in the basal ganglia may contribute to visuospatial dysfunction and slowed cognitive processing [153,155]. Moreover, mutations of mitochondrial DNA under oxidative stress across multiple brain regions are linked to attentional, mood, motor, and cognitive impairments [153,156]. Further mitochondrial defects, including impaired electron transport, loss of membrane potential, and oxidative stress in the cortex and basal ganglia, are associated with executive dysfunction, impaired problem solving, and multitasking difficulties [153]. Intriguingly, α-syn contains a non-canonical mitochondrial targeting sequence and it has been found to affect mitochondrial structure and function [157].

#### 3.2.3. Relationship with Parkinson’s Disease Dementia (PDD)

As PD progresses, many patients experience a transition from motor symptoms to significant cognitive decline, leading to the development of PDD, which is characterized by prominent deficits in executive functions, attention, and visuospatial abilities, often accompanied by fluctuations in cognition and visual hallucinations [158,159]. According to research, the risk of developing PDD increases with the duration and severity of PD. A longitudinal study found that up to 80% of patients with advanced PD may develop dementia within 20 years of diagnosis [160]. The transition from motor to cognitive symptoms often involves the propagation of α-syn pathology beyond the substantia nigra to cortical areas, contributing to the deterioration of cognitive functions. Furthermore, the presence of specific risk factors, such as an older age, the severity of motor symptoms, and the presence of depression, can further increase the likelihood of developing PDD [161].

### 3.3. Amyotrophic Lateral Sclerosis

#### 3.3.1. Cognitive Characteristics

ALS is a progressive neurodegenerative condition characterized by the loss of motor neurons in the brain and spinal cord, leading to skeletal muscle weakness, atrophy, and ultimately paralysis, severely impacting the ability to perform daily activities [2]. While ALS primarily affects voluntary muscle control, it can also present with cognitive and behavioral changes, such as executive dysfunction and personality alterations, that complicate the clinical picture. A key feature is executive dysfunction, which affects a person’s ability to plan, organize, and manage tasks. Patients may struggle with multitasking, decision-making, and adapting to new situations, often resulting in a marked decline in their daily functioning and independence [162]. Behavioral changes in ALS can be severe and variable. Apathy is a common symptom, characterized by diminished motivation, emotional retreat, and disinterest in previously enjoyed activities [163]. This lack of initiative can be particularly challenging for caregivers, who may interpret these changes as symptoms of depression or simply a lack of motivation. Disinhibition, or socially inappropriate behavior, is also frequently observed. Patients may demonstrate impulsivity, loss of social decorum, and a tendency to act without considering the consequences, which can lead to strained relationships and increased social isolation [164]. Research indicates that cognitive impairments in ALS are often mild at first but can progress to more severe dysfunction, affecting language and social cognition [165,166]. These cognitive deficits may not be recognized until advanced stages, highlighting the need for routine cognitive screening in ALS patients. A thorough assessment of cognitive and behavioral changes can significantly improve patient care by guiding supportive interventions aimed at improving quality of life for both patients and their families [167,168].

#### 3.3.2. Molecular Mechanisms Underlying Cognitive Impairments

The exact mechanisms underpinning cognitive impairments in ALS are not widely understood, but several hypotheses have been suggested [169]. One of them proposed that the degenerative process impacts not only motor neurons but also other regions in the CNS, such as the frontal and temporal lobes, which are essential for cognitive function [170]. Another hypothesis suggested that the cerebral accumulation of aberrant proteins, mainly TDP-43, contributes to cognitive dysfunction in ALS [171]. In addition to TDP-43, other proteins such as FUS and SOD1 have been implicated, highlighting the complexity of its molecular underpinnings. In ALS, PQC systems become overwhelmed or dysfunctional, reducing their ability to prevent the aggregation of TDP-43, FUS, and mutant SOD1, thereby promoting proteotoxic stress [23]. Increasing the expression of molecular chaperones can prevent protein aggregation and protect neurons in experimental models [172,173,174].

Furthermore, neuroinflammation and oxidative stress act as key amplifying mechanisms in ALS-related cognitive impairment [8]. Activated microglia and astrocytes can contribute to neuronal damage through the release of pro-inflammatory cytokines and ROS, further exacerbating neurodegeneration and impairing protein clearance mechanisms [175]. Dysfunctional microglia show a reduced capacity to degrade misfolded proteins like TDP-43 and FUS, thus facilitating their extracellular spread which ultimately results in neuronal degeneration and synaptic dysfunction within frontal and temporal regions, contributing to deficits in executive function, attention, and decision-making [165,176,177]. The chronic inflammatory response not only accelerates neuronal loss but also impairs synaptic plasticity, thereby limiting cognitive processes such as learning and memory [178].

Mitochondrial dysfunction further reinforces cognitive network vulnerability in ALS. Alterations in mitochondrial bioenergetics, dynamics, and axonal transport result in ATP depletion and increased ROS generation, affecting neuronal survival not only in motor neurons but also in cortical regions involved in cognition [3,179]. Mice carrying TDP-43 mutations exhibit mitochondrial dysfunction, neuronal loss, and significant cognitive deficits, particularly in frontal-cortex-dependent functions [165,180]. Notably, inhibiting the cytoplasmic accumulation of TDP-43 restores mitochondrial function, prevents neuronal loss and alleviates both motor and cognitive impairments, suggesting a direct causal relationship [180]. Similarly, ALS-mouse models expressing human *FUS* mutations show cognitive deficits associated with synaptic dysfunction, accompanied by a disruption in protein homeostasis and mitochondrial functioning [165,181].

## 4. Clinical Biomarkers Linking Protein Misfolding to Cognitive Decline

Cognitive impairment in NDs reflects the downstream clinical manifestation of molecular and network-level dysfunction. Integrating fluid, imaging, and neuropsychological biomarkers is essential to bridge misfolded protein pathology with measurable changes in cognition and disease progression. However, such integration is not always easy and requires a careful interpretation of data across different modalities [182].

### 4.1. Fluid Biomarkers of Protein Misfolding and Neurodegeneration

Fluid biomarkers offer minimally invasive measures of underlying pathology and neurodegeneration [183]. Classical cerebrospinal fluid (CSF) indicators, such as decreased Aβ42 and elevated p-tau, are strongly associated with AD pathology, episodic memory impairment, and progression from MCI to dementia. For instance, plasma and CSF levels of p-tau181 and p-tau231 distinguish AD patients, MCI individuals, and healthy controls and they correlate with cognitive status across disease stages [184]. Neurofilament light chain (NfL), a marker of neuroaxonal degeneration that can be detected in both CSF and blood, reflects axonal injury and is elevated across multiple NDs. Higher NfL levels correlate with greater cognitive decline and disease severity, although it is a generally non-specific marker of neuronal damage that can increase in different conditions other than AD [185]. Emerging fluid biomarkers include glial fibrillary acidic protein (GFAP) and markers of synaptic dysfunction (e.g., neurogranin), which may predict cognitive decline independently from traditional amyloid/tau markers. Plasma GFAP and p-tau181, in particular, have shown to predict future brain atrophy and longitudinal cognitive decline even in cognitively unimpaired individuals, suggesting a potential role in early risk stratification [186]. In one study, increased levels of GFAP in serum were found in ALS patients, although they were inversely linked with cognitive scores [187].

CSF studies in PD indicate that ratios of phosphorylated to total α-syn and elevated CSF NfL are associated with early deficits in memory, attention, and executive function, suggesting their potential utility in identifying PD patients at higher risk for cognitive impairment. However, the findings are sometimes heterogenous and need further validations in larger cohorts [188].

### 4.2. Neuroimaging Correlates of Network Dysfunction

Neuroimaging biomarkers improve fluid measurements by capturing structural and functional network alterations that correlates with cognitive symptoms. Structural magnetic resonance imaging (MRI) reveals disease-specific patterns of atrophy, such as hippocampal atrophy in AD, frontostriatal changes in PD, and frontotemporal atrophy in ALS, which are all associated with the main cognitive phenotype. Functional MRI studies demonstrate that altered connectivity within large-scale networks (e.g., default mode and executive control networks) may be detectable before overt atrophy, providing sensitive imaging markers of early disease progression and possibly preclinical stages [189]. Positron emission tomography (PET) imaging further allows the in vivo visualization of amyloid and tau pathology in AD, facilitating the correlation of molecular aggregates with network dysfunction and cognitive deficits. Emerging multimodal imaging combining structural and functional modalities increasingly enhances diagnostic and prognostic power, particularly when integrated with fluid biomarkers, even if standardization across centers remains a challenge [190].

### 4.3. Neuropsychological Biomarkers and Domain-Specific Cognitive Mapping

Neuropsychological assessment remains an essential tool for mapping cognitive decline to specific neural substrates. A decline in episodic memory, executive control, attention, and visuospatial ability correlates with underlying pathology as reflected by fluid and imaging biomarkers. In clinically normal adults, baseline AD biomarkers, such as CSF p-tau/Aβ42 ratio and hippocampal atrophy, have been associated with subsequent self-reported declines in memory, attention, and spatial navigation, indicating that subtle cognitive changes can be forecasted by biomarker burden even before clinical impairment is formally diagnosed [191]. Importantly, neuropsychological profiles may vary across individuals depending on the cognitive reserve, educational background, and comorbidities, complicating the interpretation of test results in longitudinal studies [192].

### 4.4. Integrative Multimodal Framework

A multimodal biomarker strategy that combines fluid measures (Aβ42, p-tau, NfL, GFAP), imaging (MRI, PET, functional connectivity), and neuropsychological assessment provides the most comprehensive approach to linking molecular pathology with cognitive outcomes. This integrative approach promotes early diagnosis, patient stratification, the monitoring of disease progression, and evaluation of mechanism-based interventions in NDs, although implementation in routine clinical practice is still limited [193]. Emerging research suggests that machine learning models incorporating fluid biomarkers with imaging and cognitive data may further enhance diagnostic accuracy and prognosis prediction, particularly in prodromal stages, but external validation and the harmonization of datasets are still needed [194].

## 5. Management and Treatment of Cognitive Deficits

The management of cognitive disorders requires a multifaceted approach that combines pharmacological treatments, non-pharmacological interventions, and, increasingly, experimental therapies. Each of these strategies addresses distinct aspects of cognitive decline and symptom management, aiming to improve the quality of life, slow disease progression, and provide symptomatic relief. Here, we examine current pharmacological options, non-pharmacological interventions, and promising experimental treatments for managing cognitive deficits.

### 5.1. Pharmacological Treatments

Pharmacological options are widely used to manage cognitive symptoms in AD and PD whereas ALS has fewer options for cognitive intervention due to its unique pathology and complex symptom presentation (Table 1). In AD, acetylcholinesterase inhibitors like donepezil and rivastigmine help alleviate cognitive symptoms by increasing acetylcholine levels in the brain, providing symptomatic relief for memory and attention deficits. Memantine, an N-methyl-d-aspartate (NMDA) receptor antagonist, is frequently used in moderate-to-severe cases to reduce excitotoxicity linked to disease progression [195,196]. Recently, three monoclonal antibodies, aducanumab, lecanemab and donanemab, were launched for AD treatment with the aim of removing Aβ protein deposits from the brain parenchyma and changing clinical trajectories. While aducanumab was withdrawn from the market in 2024 due to insufficient evidence of its clinical efficacy and concerns over its safety, clinical trials with lecanemab and donanemab resulted in a substantial decrease in Aβ deposits in the brain associated with statistically significant results in cognitive and functional performances [197]. Beyond these, emerging therapies targeting protein misfolding are under investigation. Among them, phase III clinical studies are already underway for ALZ-801, the first oral medication intended to modify the progression of AD. This drug targets an early form of Aβ and has been evaluated in apolipoprotein E (*APOE*) ε4/ε4 homozygous patients with early AD, showing a lower risk of side effects [198]. Regarding tau, bepranemab, an investigational monoclonal antibody targeting this protein currently in phase II clinical trial, has been shown to slow tau pathology and cognitive decline in patients with prodromal to mild AD [199].

Levodopa remains the primary drug for managing motor symptoms in PD, though it has a modest effect on cognitive impairment [200]. Currently, rivastigmine is the only approved medication for PDD, showing significant benefits in cognitive and functional measures along with potential reductions in visual hallucinations [201]. Similar to AD, several immunotherapies targeting misfolded α-syn are in clinical trials for PD. Among them, prasinezumab is the first monoclonal antibody that specifically targets aggregated α-syn for degradation with the aim of protecting neurons, interfering with the prion-like spreading of misfolded α-syn, and slowing disease progression [202]. For PD-related neuropsychiatric symptoms, anti-psychotic medications such as clozapine and pimavanserin are used to target serotonin and dopamine receptors, thereby alleviating behavioral symptoms without exacerbating motor deficits [203,204].

The Food and Drug Administration (FDA) has approved two drugs for the treatment of all ALS patients: the glutamate-release inhibitor riluzole and edaravone, a free radical scavenger and potent antioxidant [205,206,207,208]. Tofersen, a novel antisense oligonucleotide drug, was recently approved for people with SOD1-related genetic ALS [209]. At present, there are no specific medications approved to address cognitive impairment in ALS and cholinesterase inhibitors do not appear to modulate MCI risk in ALS [166].

#### Targeted Protein Degradation Technologies

Targeted protein degradation (TPD) methods have recently emerged as promising approach for removing specific disease-causing proteins by activating endogenous degradation pathways via UPS or autophagy–lysosome machinery [210]. Among the different TPD technologies, one of the most extensively explored and clinically advanced is the use of PROteolysis-TArgeting Chimeras (PROTACs) [211]. PROTACs are heterobifunctional molecules that recruit an E3 ubiquitin ligase to a target protein, leading to its ubiquitination and subsequent degradation by the proteasome [212].

Regarding AD, some PROTACs have been developed to specifically degrade hyperphosphorylated tau [213,214,215]. A study identified Keap1 as a promising substrate adaptor protein for ubiquitin E3 ligase in the degradation of intracellular tau [214]. Wang and collaborators developed a small molecule PROTAC that simultaneously recruited tau and E3-ligase (VHL). This PROTAC efficiently promoted the clearance of tau and improved cognitive dysfunction in a mouse model of AD [213]. In PD, the majority of PROTACs employed ligands for von Hippel–Lindau (VHL) or cereblon (CRBN) E3 for specific α-syn degradation [216,217,218]. Recently, a single amino-acid-based PROTAC, using arginine as the E3 ligand, has been developed. This approach offers several advantages over previous ones, such as reduced size and occurrence without toxicity, and is recognized by highly conserved E3 ligases [219]. For ALS, some authors developed different TDP-43 PROTACs with variable compositions of linkers that were able to effectively degrade misfolded C-TDP-43 rather than the endogenous one [220]. A recent study established a brain-penetrant DNA nanoflower technology that delivers oligonucleotide-based PROTACs capable of efficiently degrading toxic FUS proteins involved in ALS [221].

### 5.2. Non-Pharmacological Interventions

Non-pharmacological therapies provide essential support for cognitive symptoms across AD, PD, and ALS with a focus on enhancing daily functioning and quality of life. Cognitive stimulation therapy (CST) and neuropsychological training have proven beneficial in AD and are being increasingly explored in ALS [222]. For ALS patients with executive dysfunction, cognitive exercises tailored to attention, planning, and problem-solving may improve daily functioning and slow cognitive decline. Studies on structured cognitive rehabilitation in ALS indicate that it can benefit specific cognitive domains, although personalized approaches are recommended due to variability in ALS symptom presentation [223].

Occupational therapy is beneficial in all NDs, including ALS, because physical and cognitive limitations require adaptations in daily life. In ALS, therapists work closely with patients to modify tasks and use assistive technologies that compensate for motor and cognitive deficits. This can significantly reduce caregiver burden and enhance patient autonomy, supporting both cognitive and physical functions over the disease course [224].

## 6. Conclusions and Future Perspectives

AD, PD, and ALS are increasingly being recognized as disorders driven by proteostasis failure, in which the progressive accumulation of misfolded proteins destabilizes cell homeostasis and converges on synaptic dysfunction, ultimately leading to cognitive impairment [225,226]. A deeper knowledge of the intricate mechanisms underneath misfolded processes, aggregation, clearance, and intercellular propagation may offer a promising avenue for future therapeutic interventions.

A critical integrative point is the importance of regional vulnerability. The clinical heterogeneity of cognitive symptoms across NDs appears to be influenced not only by the identity of the misfolded protein but also by its anatomical location, as well as by the intrinsic metabolic and connectivity features of vulnerable neural networks. Hippocampal susceptibility in AD is responsible for episodic memory impairment, fronto-striatal circuit involvement in PD contributes to executive dysfunction, and frontotemporal degeneration in ALS is linked to behavioral and executive changes. These disease-specific cognitive patterns represent distinct anatomical appearances of a common molecular cascade [227,228].

Advances in biomarker development and imaging techniques are crucial for better understanding disease mechanisms and enabling early intervention. In vivo imaging with higher resolution may allow the real-time visualization of the formation and spread of protein aggregates [229]. Additionally, the development of more disease-specific and sensitive biomarkers will also be critical for early detection, monitoring disease progression, and assessing treatment response [230]. Improved discoveries of biomarkers will be essential for identifying preclinical or prodromal stages, when treatment may be most effective.

While numerous aggregation inhibitors have been found in vitro and in vivo, no such drug currently exists to completely prevent or reverse disease progression. Effective therapeutic strategies should not inhibit aggregation but also restore proteostasis by enhancing the activity of UPS, ALP, and chaperones [231]. Additional approaches should include preserving synaptic plasticity, regulating mitochondrial function, modulating neuroinflammation, and targeting toxic oligomeric species early in the disease progression. Interventions addressing synaptic and network dysfunction before irreversible neuronal loss may offer the greatest potential to mitigate cognitive decline, even if translating these findings into clinical practice remains challenging [232,233]. Precision medicine strategies that incorporate genetic risk, molecular profiling, and longitudinal monitoring may further improve therapeutic targeting and enable pre-symptomatic intervention. Importantly, modifying disease trajectories may necessitate combination therapies that target many pathogenic nodes simultaneously [15]. Additional research efforts should also include both drug repurposing and the identification of medications with novel mechanisms of action.

Given advancements in imaging, high-throughput screening, and omics, future research should focus on integrative, systems-oriented models that can link molecular pathology to network dynamics and domain-specific cognitive outcomes. Such approaches may facilitate the development of biomarker-guided, mechanism-based therapies applicable across NDs, but further longitudinal and cross-cohort investigations are needed. To achieve significant progress in preventing or delaying cognitive decline, coordinated, multidisciplinary teamwork and the integration of advanced technologies will be required.

## Figures and Tables

**Table 1:** Overview of pharmacological treatments targeting cognitive deficits in AD, PD, and ALS.

Disease	Drug	Mechanism	Population	Cognitive Outcome
AD	Donepezil	Inhibiting acetylcholinesterase	Mild–moderate AD patients	Improved memory and executive daily functioning
	Rivastigmine	Inhibiting acetylcholinesterase	Mild–moderate AD patients	Improved memory and executive daily functioning
	Memantine	Reducing glutamate-mediated excitotoxicity	Moderate–severe AD patients	Slowed-down cognitive decline and improved daily functioning
	Lacanemab	Inhibiting aggregation process by binding soluble Aβ protofibrils	MCI-mild AD patients	Improved memory and executive daily functioning
	Donanemab	Facilitating immune-mediated clearance of existing amyloid deposits by binding a specific pyroglutamate-modified Aβ	Early symptomatic AD patients with low-intermediate levels of tau protein	Improved memory and executive daily functioning
	ALZ-801	Inhibiting Aβ oligomer formation	*APOE* ε4/ε4 homozygous with early AD patients (phase III)	Slowed-down cognitive decline
	Bepranemab	Inhibiting tau accumulation	Prodromal-mild AD patients (phase II)	Slowed-down cognitive decline
PD	Levodopa	Replacing dopamine	PDD patients	Modest effects on cognitive impairment
	Rivastigmine	Inhibiting acetylcholinesterase	PDD patients	Improved cognitive performance and reduced visual hallucinations
	Prasinezumab	Inhibiting α-syn aggregation	Early PD patients (phase III)	Under investigation
ALS	Riluzole	Inhibiting glutamate release	ALS patients	No evidence on cognitive benefit
	Edaravone	Reducing oxidative stress	ALS patients	No evidence on cognitive benefit
	Tofersen	Degrading *SOD1* mRNA	ALS patients carrying *SOD1* mutations	No evidence on cognitive benefit

## Data Availability

No new data were created.

## Abbreviations

The following abbreviations have been used in this manuscript:

AD	Alzheimer’s disease
ALP	autophagy–lysosomal pathway
ALS	amyotrophic lateral sclerosis
APOE	apolipoprotein E
ATP	adenosine triphosphate
CSF	cerebrospinal fluid
CNS	central nervous system
CST	cognitive stimulation therapy
C9ORF72	chromosome 9 open reading frame 72
DAMPs	damage-associated molecular patterns
FDA	food and drug administration
FUS/TLS	fused in sarcoma/translocated in liposarcoma
GFAP	glial fibrillary acidic protein
Hsps	heat shock proteins
LTD	long-term depression
LTP	long-term potentiation
MCI	mild cognitive impairment
MRI	magnetic resonance imaging
NDs	neurodegenerative disorders
NfL	neurofilament light chain
NFT	neurofibrillary tangle
NLS	nuclear localization signal
NMDA	N-methyl-d-aspartate
PD	Parkinson’s disease
PDD	Parkinson’s disease dementia
PET	positron emission tomography
PQC	protein quality control
PROTACs	PROteolysis TArgeting Chimeras
PTMs	post-translational modifications
ROS	reactive oxygen species
sHsps	small heat shock proteins
SOD	superoxide dismutase
TDP-43	TAR DNA-binding protein 43
TPD	targeted protein degradation
UPS	ubiquitin-proteasome system
